# Caries Detection in Primary Teeth Using Intraoral Scanners Featuring Fluorescence: Protocol for a Diagnostic Agreement Study

**DOI:** 10.2196/51578

**Published:** 2023-12-14

**Authors:** Bree Jones, Stavroula Michou, Tong Chen, Margarita Moreno-Betancur, Nicky Kilpatrick, David Burgner, Christoph Vannahme, Mihiri Silva

**Affiliations:** 1 Murdoch Children's Research Institute Royal Children's Hospital Darley Australia; 2 Melbourne Dental School University of Melbourne Melbourne Australia; 3 Department of Odontology School of Dentistry University of Copenhagen Copenhagen Denmark; 4 3Shape TRIOS A/S Copenhagen Denmark; 5 Clinical Epidemiology & Biostatistics Unit Murdoch Children's Research Institute Royal Children's Hospital Melbourne Australia; 6 Department of Paediatrics University of Melbourne Melbourne Australia

**Keywords:** dental caries, diagnosis, oral, technology, dental, image interpretation, computer-assisted, imaging, 3D, quantitative light-induced fluorescence, diagnostic agreement, intra oral scanners, oral health, teeth, 3D model, color, fluorescence, intraoral scanner, device, dentistry

## Abstract

**Background:**

Digital methods that enable early caries identification can streamline data collection in research and optimize dental examinations for young children. Intraoral scanners are devices used for creating 3D models of teeth in dentistry and are being rapidly adopted into clinical workflows. Integrating fluorescence technology into scanner hardware can support early caries detection. However, the performance of caries detection methods using 3D models featuring color and fluorescence in primary teeth is unknown.

**Objective:**

This study aims to assess the diagnostic agreement between visual examination (VE), on-screen assessment of 3D models in approximate natural colors with and without fluorescence, and application of an automated caries scoring system to the 3D models with fluorescence for caries detection in primary teeth.

**Methods:**

The study sample will be drawn from eligible participants in a randomized controlled trial at the Royal Children’s Hospital, Melbourne, Australia, where a dental assessment was conducted, including VE using the International Caries Detection and Assessment System (ICDAS) and intraoral scan using the TRIOS 4 (3Shape TRIOS A/S). Participant clinical records will be collected, and all records meeting eligibility criteria will be subject to an on-screen assessment of 3D models by 4 dental practitioners. First, all primary tooth surfaces will be examined for caries based on 3D geometry and color, using a merged ICDAS index. Second, the on-screen assessment of 3D models will include fluorescence, where caries will be classified using a merged ICDAS index that has been modified to incorporate fluorescence criteria. After 4 weeks, all examiners will repeat the on-screen assessment for all 3D models. Finally, an automated caries scoring system will be used to classify caries on primary occlusal surfaces. The agreement in the total number of caries detected per person between methods will be assessed using a Bland-Altman analysis and intraclass correlation coefficients. At a tooth surface level, agreement between methods will be estimated using multilevel models to account for the clustering of dental data.

**Results:**

Automated caries scoring of 3D models was completed as of October 2023, with the publication of results expected by July 2024. On-screen assessment has commenced, with the expected completion of scoring and data analysis by March 2024. Results will be disseminated by the end of 2024.

**Conclusions:**

The study outcomes may inform new practices that use digital models to facilitate dental assessments. Novel approaches that enable remote dental examination without compromising the accuracy of VE have wide applications in the research environment, clinical practice, and the provision of teledentistry.

**Trial Registration:**

Australian New Zealand Clinical Trials Registry ACTRN12622001237774; https://www.anzctr.org.au/Trial/Registration/TrialReview.aspx?id=384632

**International Registered Report Identifier (IRRID):**

DERR1-10.2196/51578

## Introduction

### Background

Dental caries is the most prevalent chronic disease in childhood [1]. It causes mineral loss within the dental hard tissue, which results in visual changes in the enamel in the initial stages [2]. With increasing severity, demineralization advances into the dentine-pulp complex, and a cavity can develop [2]. More severe caries can result in pain, difficulty eating, and infection, and its management is more invasive and costly from an individual and public health perspective. In contrast, managing early caries is noninvasive, and damage to the tooth surface is reversible [3]. Epidemiological research should use methods that enable early disease detection to better target caries prevention and timely intervention programs during the early years.

### Dental Caries Detection in Epidemiological Research

In epidemiological research, visual examination (VE) is the standard method for detecting dental caries on visible tooth surfaces. VE can be subjective, particularly for early disease detection and monitoring. Validated indices, such as the International Caries Detection and Assessment System (ICDAS), improve the accuracy of VE for early caries detection [4-6]. ICDAS classifies caries based on severity, and a strong correlation exists between ICDAS and histological caries depth [7,8]. Studies using ICDAS report good inter- and intraexaminer reliability. Training and calibration improve examiner accuracy and reliability and are essential for standardizing examiners [9-11].

Epidemiological research, particularly multisite studies, requires the presence of multiple qualified dental examiners to undertake VE at the time of data collection, which is time and resource intensive. Digital photography has been suggested as an alternative to VE in these settings, where images are taken and assessed by trained and calibrated examiners. Boye et al [12] demonstrated high examiner agreement (weighted kappa, κ_w_>0.9) between VE and remote assessment of 2D images. High accuracy for caries detection has been reported using 2D images compared to a gold standard examiner; however, the threshold used for disease detection in these studies is mostly tooth cavitation. Such thresholds cannot inform and drive early disease management strategies. Novel methods that facilitate the remote assessment of dentitions without compromising accuracy relative to VE for early disease detection would have broad applications in the research setting and beyond. Intraoral scanning technology provides such a method, yet its performance for caries detection in children’s primary teeth has yet to be established.

### Intraoral Scanners for Dental Caries Detection

Intraoral scanners (IOSs) are handheld devices that create 3D digital models of the teeth and surrounding structures [13]. Digital representation of the teeth is achieved using specialized hardware (to scan the teeth) and software (to combine the data received and create 3D models). Scanners shine light onto a tooth region, and the distance between the area of interest and the scanner sensor is calculated via optical triangulation, confocal imaging, or active wave front sampling [14,15]. Numerous data points regarding tissue geometry are generated and used to construct a 3D model [14]. Red, green, and blue signals received from the tissues are applied to the model as color texture. Recently, light-induced fluorescence technology has been incorporated into the hardware of a commercially available IOS to enhance early caries detection (TRIOS 4 and 5) [16].

Light fluorescence technology is suitable for detecting and quantifying early carious lesions [17]. Exposure to blue-violet wavelength light causes sound dental tissue to auto-fluoresce green and demineralized carious dental tissue to appear darker [17]. The diagnostic capability of light fluorescence is enhanced because porphyrins, a metabolite of bacteria associated with dental caries, emit red fluorescence when exposed to light at this wavelength [18].

The interpretation of light fluorescence can be subjective, as it depends on the skill and experience of the dental practitioner. A study investigating occlusal caries detection of permanent teeth with and without fluorescence found no difference in accuracy between on-screen assessment and VE, suggesting that on-screen assessment of 3D models could be used for early caries detection and as a potential alternative to VE [19]. To our knowledge, there are no studies that have investigated the validity of on-screen assessment of 3D models for caries detection in primary teeth.

Recent advances have seen the development of computational methods as a more objective measure to quantify dental caries and support clinical diagnosis. TRIOS has developed an automated caries scoring system for their software that can automatically classify caries on occlusal surfaces based on color and fluorescence changes (TRIOS Patient Monitoring [TPM]; version 2.3; 3Shape TRIOS A/S) [16]. When comparing the diagnostic performance as quantified by the area under the curve (AUC), early in vitro and in vivo validation studies suggest that the automated score is comparable to VE at the early (AUC VE 0.71 vs 0.76) and moderate disease thresholds (AUC VE 0.90 vs 0.90), based on a histological reference standard for permanent teeth [20]. A separate investigation into the validity of this algorithm in primary teeth concluded it had comparable performance (AUC 0.88) to VE (AUC 0.96) for caries detection of primary tooth occlusal surfaces [21]. The latter was a small in vitro feasibility study; consequently, results should be interpreted cautiously and not generalized to the clinical setting. The in vivo performance of automated assessment of 3D models for caries detection in primary teeth has yet to be established.

One of the problems with investigating early caries detection methods in realistic settings is the choice of a reference standard for method comparison, as histological or operative references are rarely feasible or ethical. It is argued that when VE alone is used as a gold standard, measures of diagnostic accuracy (sensitivity and specificity) may overestimate the true accuracy of the caries detection method under investigation [22,23]. Instead, reporting on the diagnostic agreement has been suggested to be more appropriate. Diagnostic agreement quantifies the similarity between methods in detecting the outcome of interest to be used interchangeably [24].

This study describes the protocol and statistical analysis plan for determining the diagnostic agreement between VE, on-screen, and automated caries detection methods using 3D models in children.

## Methods

### Aims and Objectives

The specific study objectives are to, first, determine and compare the diagnostic agreement between VE and on-screen visual assessment of 3D models in approximate natural colors with and without fluorescence to detect and classify carious lesions on visible tooth surfaces in primary teeth. The second objective is to determine the diagnostic agreement between VE and the automated caries scoring system for detecting and classifying occlusal carious lesions in primary teeth. Finally, the third objective is to explore the impact of caries threshold, tooth surface examined (smooth vs occlusal), and enamel defects’ presence on the reported agreement estimates between caries detection methods.

### Study Design

This is a diagnostic agreement study, as VE is not considered a gold standard in this setting [23]. Dental caries in this study is defined as primary coronal caries. Data sources (age at dental visit, sex, VE data, and 3D models) will be retrospectively sourced from a sample of children who underwent VE using the ICDAS and intraoral scanning with the TRIOS 4 to produce 3D digital models.

**Figure 1:**
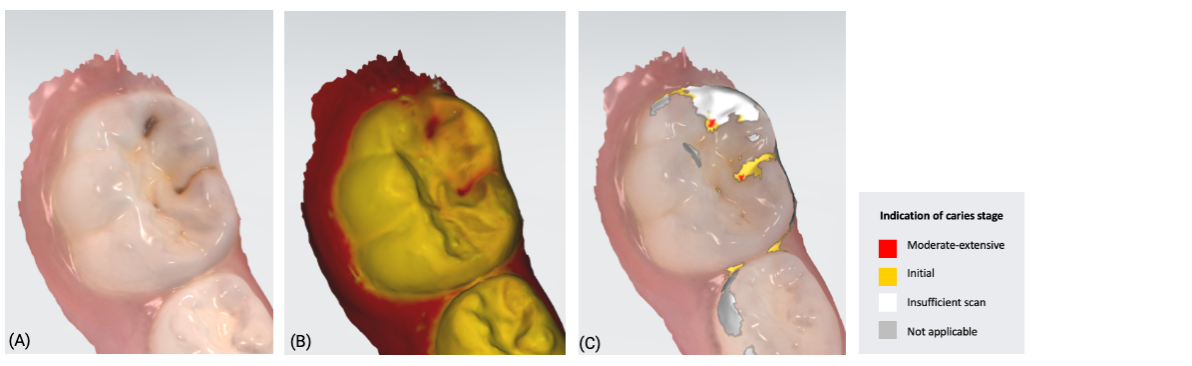
Caries detection methods based on 3D models obtained using an intraoral scanner.

The 3D models for each eligible participant will be subject to an on-screen assessment by 4 qualified dental practitioners. Each practitioner will examine all 3D models at 2 separate time points. An on-screen assessment of 3D models based on tissue geometry and approximate natural colors (excitation to visible white light) will take place first (Figure 1A), followed by an on-screen assessment with the addition of fluorescence texture (excitation to light at 415 nm; Figure 1B). An interval of at least 4 weeks will follow to minimize recall bias before the on-screen assessment process is replicated. Occlusal surfaces of primary molars will be examined using the automated caries scoring system at a single time point only (Figure 1C). Examiners will be blinded to all clinical examination data and automated caries scores when scoring the 3D scans.

### Study Setting and Participants

The study sample will be drawn from eligible participants in an existing randomized controlled trial, the Melbourne Infant Study Bacille Calmette Guérin (BCG) for Allergy & Infection Reduction (MISBAIR; ClinicalTrials.gov NCT01906853; for a complete description of the trial, please refer to the MISBAIR study protocol) [25]. English-speaking immunocompetent pregnant women scheduled for birth at hospitals servicing Werribee, Ballarat, Geelong, and Melbourne in Victoria, Australia, from mid-2013 to mid- to late 2016 provided consent for their infants to participate in a longitudinal and randomized controlled trial to determine if BCG immunization within the first 10 days of life prevents the development of allergy and infection in early childhood. Infants were excluded if they had any conditions at birth that would contraindicate the use of live vaccines. The MISBAIR trial included a 5-year-old study visit where a clinical assessment (including dental assessment) was undertaken (219/536, 40.9% remaining MISBAIR participants). All study visits occurred between January 31, 2021, and March 31, 2022, at the Murdoch Children’s Research Institute in Melbourne.

### Participant Eligibility Criteria

MISBAIR participant data will be included for analysis in this study if participants meet the following criteria: (1) attended the 5-year-old study visit for MISBAIR (target age 4-6 years), (2) completed of a VE during the 5-year-old study visit, (3) acquired an intraoral scan at the same time as the VE, and (4) provided informed consent for the analysis of dental data and on-screen assessment of 3D models.

### Tooth Surface Inclusion Criteria

For each participant, a tooth surface will be eligible for on-screen assessment if it (1) has been examined as part of the dental exam, (2) is from a primary tooth, and (3) is visible on the 3D model.

### Tooth Surface Exclusion Criteria

Tooth surfaces will be excluded from on-screen assessment if (1) they have a sealant, direct or indirect restoration; (2) the tooth surface visibility on the 3D model is impeded by calculus, debris, or plaque; and (3) less than one-third of the surface is visible due to insufficient or missing scan data.

Tooth surfaces will be excluded from the application of the automated caries scoring system if they (1) have a sealant, direct or indirect restoration; (2) have an enamel defect; (3) are not a primary molar occlusal surface; and (4) less than one-third of the surface is visible due to insufficient or missing scan data.

### Dental Examination Protocol at the 5-Year-Old Study Visit

#### VE Protocol

VEs occurred with participants in a semisupine position on an adjustable examination bed. The examiner stood to the side of, or behind, the participant. A portable LED light (220 lumens NÄVLINGE clamp spotlight IKEA) and a dental mirror were used and positioned to provide maximum illumination and visibility of the oral cavity without magnification. Cotton rolls and swabs were used to dry teeth and remove plaque. Compressed air was not used. Universal infection control standards and additional COVID-19 precautions were adhered to for all examinations.

Dental examinations were conducted by trained and calibrated registered dental practitioners (BJ and Jennifer Copley) with experience and prior training in using the ICDAS index in research settings. Examiner BJ is an oral health therapist with 13 years of clinical experience, and examiner Jennifer Copley is an oral health therapist with 5 years of clinical experience. Before commencing VEs, both examiners had participated in the web-based ICDAS and International Caries Classification and Management System (ICCMS) e-learning modules [26]. Additionally, a senior researcher and pediatric dentist (MS) with experience in conducting surveys using ICDAS and enamel defect indices facilitated a presentation on ICDAS using the ICDAS or ICCMS examiner training materials and training materials for the use of a modified molar incisor hypomineralization (MIH) index to assess for enamel defects (with permission from the author) [27]. A 2-hour calibration session on the application and interpretation of the indices, followed by a computer-based calibration exercise, took place. The computer-based calibration exercises were repeated until both examiners reached an intra- and interexaminer agreement of κ_w_>0.8.

Dental caries experience was recorded for each tooth surface using the 2-digit ICDAS criteria [28]. Code 01 could not be scored for smooth surfaces due to a lack of compressed air. Enamel defects were recorded using a modified MIH index describing each surface’s clinical presentation and the extent of the defect [29]. All tooth surfaces were examined using a systematic approach. The score given was the most severe of the 2 clinical presentations if multiple lesions were present on the same tooth’s surface.

All dental examination data were collected and managed using Research Electronic Data Capture (REDCap; Vanderbilt University) tool hosted at Murdoch Children’s Research Institute [30,31]. REDCap is a secure, web-based software platform designed to support data capture for research studies.

#### Intraoral Scanning

Immediately following the VE, intraoral scanning took place using the TRIOS 4 scanner. This generation scanner has a high accuracy and fast acquisition time relative to other scanners on the market for complete arch scanning [32]. It also has specialized fluorescence technology built into its hardware which exposes teeth to 415-nm light during scanning. Light emitted from the teeth passes through a filter in the scanner (which filters out blue light) to be received by the scanner sensor. The fluorescence signal overlays onto the 3D models as green fluorescence and red fluorescence texture, aided by commercial software (TPM; version 2.3, Dental Desktop and TRIOS; 3Shape TRIOS A/S).

The clinical examiners were trained to use the 3Shape TRIOS software interface and intraoral scanning protocols. Before scanning, the dental lamp was switched off, and the window blinds were closed to limit external light during the scanning process as per manufacturer instructions [33]. Teeth were dried with gauze or cotton rolls, and the manufacturer-recommended scanning strategy was followed. The scanning procedure was considered adequate when the software had obtained sufficient information about tooth color and fluorescence for the region of interest. If the scan was incomplete, the reasons why the scanning procedure was inadequate were documented. The reasons for insufficient scan data included the scanner tip being too big to accommodate, gag reflex, and behavioral or developmental issues that limited cooperation with the scanning procedure. Both complete and incomplete 3D models were saved with the participant's unique identifier in the dental desktop software.

### Dental Examination Protocol for 3D Models Obtained From an IOS

#### On-Screen Assessment

3D models will be examined in postprocessed formats on a laptop with a 15-inch monitor (Alienware, DELL). On-screen assessment in approximate natural colors and featuring fluorescence will occur using a noncommercial software (developed by 3Shape) that allows for visualization of the 3D models similar to the commercially available TPM software but permits faster examination of multiple models, making the viewing process more efficient for this study. 3D models will be viewed in rooms with natural lighting following a standard operating procedure. Examination periods will be at most 3 hours without breaks, and each 3D model will be viewed for less than 15 minutes to avoid examiner fatigue. The viewing time for each scan will be recorded.

Each model will be viewed first with color and then without color to inspect tissue geometry and possible surface cavitation. Dental caries experience for on-screen assessment of 3D models in color will be recorded using a merged ICDAS index. Codes 01 and 02 will be combined as “initial caries” because the 3D models cannot be dried, and codes 05 and 06 will be combined as “extensive caries” as discriminating between these latter categories is not of interest for this study (Table 1). These merged ICDAS categories still provide sufficient information about caries depth to support clinical decision-making [8,9,34].

**Table 1:** Caries classification criteria for on-screen assessment of 3D models obtained with TRIOS intraoral scanners.

Caries extent	Merged ICDAS index: on-screen assessment—color		Merged modified ICDAS index: on-screen assessment—fluorescence	
Sound (ICDAS 0)	Sound tooth surfaces which show no evidence of visible caries	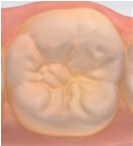	Sound surfaces (yellow-green fluorescence) with no visible fluorescence change (no red fluorescence)	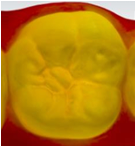
Initial (ICDAS 1-2)	First or distinct visual changes in enamel seen as carious opacity or visible discoloration (white spot lesion and brown carious discoloration) not consistent with clinical appearance of sound enamel and which show no evidence of surface break down or underlying dentine shadowing	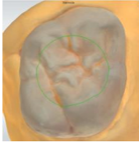	First or distinct visual changes in enamel seen with orange-red fluorescence and altered green fluorescence not consistent with the appearance of sound enamel and which show no evidence of surface breakdown or underlying red fluorescence from dentine	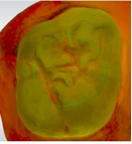
Moderate (ICDAS 3)	A white or brown spot lesion with localized microcavitation of enamel without visible dentine exposure	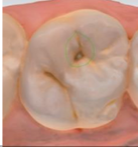	Lesion with localized enamel breakdown with a distinct fluorescence change (usually intense red and reduced green), without visible dentine exposure. Usually, poorly delineated fluorescence change is seen	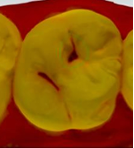
Moderate (ICDAS 4)	Obviously discolored dentine or dark shadow visible through apparently intact or microcavitated enamel, which has originated on the surface being evaluated	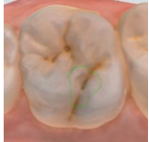	Lesion with or without enamel breakdown and poorly delineated distinct fluorescence change (intense red fluorescence and significantly reduced green fluorescence which appears almost black), deriving from dentine	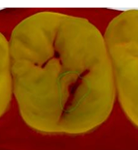
Extensive (ICDAS 5-6)	A distinct cavity in opaque or discolored enamel with visible dentine	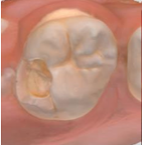	Distinct cavity with visible fluorescence changes and exposed dentine	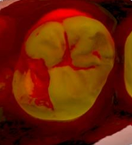

3D models will then be viewed with the addition of fluorescence texture and dental caries will be recorded using a merged ICDAS index previously described by Ferreira Zandoná et al [35], where each scoring criteria has been modified to incorporate fluorescence features (Table 1). This index enables a consistent approach to classifying carious lesions using fluorescence and has been previously validated for the on-screen assessment of 3D models [19]. If multiple lesions are present on the same tooth’s surface, the caries classification will be the more severe of the 2 clinical presentations, per VE protocol. Data will be recorded using REDCap.

A total of 4 examiners will undertake on-screen assessment. Before commencing on-screen assessment, all examiners will undergo a 2-hour training workshop followed by calibration exercises. Training will focus on applying a merged and modified ICDAS criteria to a data set of 3D models with fluorescence. A dentist and expert in using modified ICDAS to interpret 3D models (SM) will facilitate training. To establish interrater agreement, each examiner will independently undertake an on-screen assessment of a training-calibration data set of 200 examination sites. This data set includes carious lesions and sound surfaces which have been validated histologically as part of a previous study [16,20]. Examiners will repeat this calibration exercise 2 weeks after the initial assessment to establish an intrarater agreement. Examiners will repeat the calibration process until the agreement exceeds κ_w_>0.7.

#### Automated Caries Scoring System

The automated caries scoring system will be applied to the occlusal surfaces of primary teeth and visualized using the commercially available software (TPM version 2.3; 3Shape TRIOS A/S) on a laptop with a 15-inch monitor (Alienware, DELL). Using a predefined logistic regression function, this software identifies a healthy reference standard per tooth and compares regions of interest to the healthy reference, to quantify fluorescence changes [16]. The automated system classifies a region of interest as caries based on the predefined cut offs which have been validated using permanent tooth histology as a reference standard, to indicate relative caries depth. The software classifies a surface as sound, initial caries (ICDAS 01/02) or moderate-extensive caries (ICDAS>03), and this is indicated on the 3D models as a yellow or red overlay respectively (Figure 1). The automated classification will be recorded using REDCap.

### Sample Size

This is a retrospective study drawing all eligible participants from the MISBAIR trial who underwent the dental assessment at their 5-year-old study visit.

### Outcome Measures

For agreement analyses, ICDAS and modified ICDAS codes will be collapsed for each surface into four categories labeled sound surface (0), initial caries (1), moderate (2), and extensive caries (3). A binary dental caries variable will be created for each surface, and the detection threshold will be initial caries (0 vs 1, 2, and 3).

### Statistical Analysis

Data checking and cleaning will take place before analysis. Descriptive statistics for patient characteristics such as age, sex, caries prevalence, and the proportion of children with enamel defects will be reported. The total number of tooth sites included in the analysis will be reported.

Descriptive characteristics for the rater population will be reported, including qualification, clinical background, years of experience, and training results for each rater. Interrater and intrarater reliability for on-screen assessment of 3D models based on color and fluorescence using the intraclass correlation coefficient will be reported for all examiners. These estimates will be obtained from a multilevel model fitted to the surface-level data, accounting clustering (see below), and zero inflation.

To assess agreement among methods, we will undertake both surface-level and individual-level analyses. At the surface level, caries data naturally have 3 levels with surfaces clustered within a tooth, which are clustered within an individual. Therefore, for this analysis, we will use multilevel logistic regression to estimate method agreement while accounting for clustering for the tooth. Specifically, separate multilevel models will be fitted to compare VE with each of the alternative methods by including the variable method (VE vs on-screen assessment of 3D models in approximate natural colors, on-screen assessment of 3D models in approximate natural colors featuring fluorescence, and automated system) as the fixed effect. The coefficient estimates for the variable method will be used to indicate the level of agreement. The multilevel model will be fitted with a zero-inflated binomial distribution.

At the individual level, a numerical variable representing the number of lesions per individual will be generated and analyzed with 2 approaches. First, Bland-Altman method agreement analysis will be performed using a regression approach. Specifically, a model will be constructed to regress the difference between each pair of methods on their average [36]. The analysis will be repeated for each possible pair of methods. Second, for each possible pair of methods, an intraclass correlation coefficient will be estimated from a multilevel model fitted to the paired data consisting of the 2 measures for each individual obtained from the 2 methods, fitted with a zero-inflated Poisson distribution.

A sensitivity analysis will explore the impact of the diagnostic threshold used on agreement estimates. Analyses will be repeated with the caries data dichotomized at alternative thresholds: the moderate and extensive thresholds. In the analysis of the moderate threshold, sound and initial surfaces will be labeled caries absent (0) and all remaining categories (moderate-extensive) will be labeled caries present (1). In the analysis of the extensive threshold, sound, initial, and moderate caries will be combined and labeled as caries absent (0) and only teeth with extensive caries will be marked as caries present (1). To evaluate if the surface location and presence of enamel defects impact the diagnostic agreement estimates, these variables will be included as additional variables in the regression models for the surface-level analyses, including interaction terms with the method. The surface location variable will have 3 levels for each visible tooth surface (occlusal, smooth, and proximal). Enamel defects will be a binary variable, defined as present or absent per person. The sensitivity analysis to compare the agreement between on-screen assessment of 3D models in approximate natural colors and on-screen assessment of 3D models in approximate natural colors featuring fluorescence will include additional variables, rater and timing, as fixed effects.

### Ethical Considerations

The MISBAIR trial has ethical and governance approval from Mercy Health Human Research Ethics Committee (HREC, No. R12-28) and Royal Children’s Hospital (RCH) HREC (HREC No. 33025). Informed consent to participate in the study and to share dental data to validate and develop intraoral scanning technology for dental health assessment was obtained from participants' parents or guardians. Participant records sourced have a unique identifier and are not reidentifiable to the dental team. This diagnostic agreement study has ethical approval from the RCH HREC (RCH HREC No. 88321). It will be conducted according to the principles outlined in the Declaration of Helsinki. This protocol has registration with the Australian New Zealand Clinical Trials Registry (ANZCTR; registration ACTRN12622001237774p).

## Results

As of October 2023, automated caries scoring of primary molar occlusal surfaces on the 3D models for 213 eligible participants has been completed. Data are currently being analyzed, with expected results to be published within 6 months’ time. On-screen assessment of 3D models based on color and fluorescence has commenced and is expected to be completed in March 2024, with results disseminated by December 2024.

The results of this study will be reported per the Guidelines for Reporting Reliability and Agreement Studies [37].

## Discussion

This protocol aims to fill a knowledge gap by describing an approach for determining the diagnostic agreement between VE using ICDAS and on-screen assessment of 3D models featuring fluorescence obtained with an IOS.

Intraoral scanning is increasingly being adopted in dentistry as it offers improved clinical workflows for patient assessment and treatment planning, particularly in the disciplines of prosthodontics, orthodontics, and periodontics [13,38,39]. The role of scanning technology in supporting clinical diagnostics is an emerging research area [19,20,40-43]. There is paucity in the literature describing on-screen assessment of 3D models as a method for caries detection.

A strength of our study is the consideration of different approaches to analyze agreement. Our use of multilevel generalized linear regression models to analyze surface-level dental caries data has several strengths. First, caries from the same individual are more correlated than those between individuals. If this clustering is ignored, the estimated variance may be misleadingly small [44]. We will use multilevel models that account for clustering. Second, the multilevel regression approach allows for estimating method agreement while accounting for variability due to the influence of the rater and timing of assessment [45]. Finally, because all eligible primary teeth surfaces will be analyzed the high proportion of sound surfaces will result in zero-inflated outcome data. We will address this by fitting a generalized linear regression framework allowing the use of a zero-inflated binomial distribution; whereas traditional agreement methods, such as the κ statistic, are limited in this context [46]. A limitation of the statistical approach is that the coefficient estimates derived from the multilevel model do not quantify absolute agreement. As an alternative analysis, we will analyze the total caries at the individual level with a regression-based Bland-Altman agreement analysis [36]. The excess of zeros and, hence, the nonnormal distribution of the data precludes the calculation of the limit of agreement estimates that are clinically interpretable. Consequently, we will use the intraclass correlation coefficient derived from a multilevel model fitted with a zero-inflated Poisson distribution, acknowledging that this is influenced by the variability in the population.

Further limitations include the retrospective study design, as multiple raters did not undertake VE over multiple time points, so we cannot account for the effects of this in the analysis. The prospective design of the onscreen assessment is a strength, as it has been designed to capture these effects.

The study results will inform whether 3D models can be used interchangeably with VE for detecting and classifying dental caries. This work serves as a proof of concept for performing dental examinations digitally. Study outcomes may change existing practices for data collection in children for epidemiological research. The findings may also provide insight into how scanners can be used more broadly in the provision of teledentistry services and to improve the access of young children to comprehensive dental examinations.

## Abbreviations

ANZCTR: Australian New Zealand Clinical Trials Registry
AUC: area under the curve
BCG: Bacille Calmette Guérin
HREC: Human Research Ethics Committee
ICCMS: International Caries Classification and Management System
ICDAS: International Caries Detection and Assessment System
IOS: intraoral scanner
MIH: molar incisor hypomineralization
MISBAIR: Melbourne Infant Study-Bacille Calmette Guérin for Allergy & Infection Reduction
RCH: Royal Children’s Hospital
REDCap: Research Electronic Data Capture
TPM: TRIOS Patient Monitoring
VE: visual examination

## Data Availability

The data sets generated and analyzed during this study are not publicly available due to consent not obtained from participants for public sharing of data but are available from the corresponding author on reasonable request.
